# The effects of breed, age, sex, and body weight on electrocardiographic parameters in military working dogs

**DOI:** 10.14202/vetworld.2020.1001-1004

**Published:** 2020-05-30

**Authors:** Wichaporn Lerdweeraphon, Surangkhana Thanwongsa, Suriya Youyod, Sermsawat Imsopa, Wootichai Kenchaiwong

**Affiliations:** 1 Applied Animal Physiology Research Unit, Faculty of Veterinary Sciences, Mahasarakham University, Thailand; 2 Faculty of Veterinary Sciences, Mahasarakham University, Thailand

**Keywords:** age, breed, dogs, electrocardiogram, sex

## Abstract

**Aim:**

This study aimed to investigate the effects of breed, sex, age, and body weight on different electrocardiographic parameters in military working dogs (Labrador Retriever and German Shepherd).

**Materials and Methods:**

Electrocardiographic recordings (paper speed = 25 mm/s and calibration = 10 mm/mV) were performed to obtain all the standard bipolar limb leads (leads I, II, and III) and unipolar augmented limb leads (leads aVR, aVL, and aVF). A total of 16 Labrador Retrievers and 14 German Shepherds were restrained manually in the right lateral recumbency without any tranquilizer or anesthetic drug. Amplitude and duration of P, QRS, and T wave, PR and QT interval, mean electrical axis, and heart rate were measured in each recording.

**Results:**

There was no significant difference in electrocardiographic parameters across breed and sex. However, QRS duration tended to alter by breed (p<0.1) in Labrador Retrievers (0.04±0.005 s), which is lower than German Shepherds (0.05±0.005 s). PR interval was influenced by sex (p<0.1). PR interval was higher in females (0.13±0.005 s) than males (0.11±0.008 s). In addition, electrocardiographic values were not significantly affected by age and body weight, except that the amplitude of R wave was statistically affected by age (p<0.05). A correlation was found between the decrease in R wave amplitude and increase in age of dogs.

**Conclusion:**

Different electrocardiographic parameters were within the normal range. A significant effect of age was seen on amplitude of R wave. However, the effect of breed, sex, and body weight was not significant on different electrocardiographic parameters in Labrador Retriever and German Shepherd dogs.

## Introduction

An electrocardiograph (ECG) is a machine used to measure electrical activity of the heart to evaluate heart rate (HR) and rhythm, conduction, and mean electrical axis (MEA) [1]. It is a widely used non-invasive tool to determine cardiac arrhythmias, conduction disturbances, and heart morphology in many domestic animals [2-6].

Large-sized dogs are at high risk of cardiac arrhythmias [7-10], particularly in breeds that have inherited cardiac arrhythmias such as German Shepherds [11,12]. Congenital cardiac defect is most common in Labrador Retrievers [13]. A previous study reported that a routine yearly ECG recording in a Labrador Retriever detected a normal sinus rhythm with first degree atrioventricular (AV) blockade and atrial dissociation [14]. In addition, large-sized dogs have enough atrial mass to induce atrial arrhythmias (AF) [15]. Effects of hot climate and exercise training might alter cardiovascular adjustments on different ECG parameters in some exotic breeds of trained dogs such as Labrador, German Shepherd, and Golden Retriever [16]. Moreover, breed differences in ECG parameters in healthy dogs have been reported, which may be due to variations in thoracic shape [17]. Variations in age were significantly associated with R wave amplitude and QRS duration in German Shepherds [18]. Body weight was also associated with variations in ECG parameters, whereas age and sex were associated with variations in HR [19].

In Thailand, giant breed dogs such as Labrador Retrievers and German Shepherds are used to perform sniffing activities and military work training, but they do not have routine ECG evaluation and monitoring. Hence, ECG changes with respect to breed, age, sex, and body weight in these dogs were not clearly understood. Thus, this study aimed to examine the effects of breed, age, sex, and body weight on ECG parameters in military working dogs.

## Materials and Methods

### Ethical approval

All procedures on animals of this study were approved by the Institutional Animal Ethics Committee, Mahasarakham University, Thailand.

### Animals

This study included 30 clinically healthy dogs of both sexes in two different breeds, Labrador Retriever (n=16) and German Shepherd (n=14), from military working dog battalion, Pak Chong District, Nakhon Ratchasima Province, Thailand. Animals’ age ranged from 1 to 8 years with body weight of 20-50 kg. Before the recording, all animals were examined carefully to screen for heart diseases.

### Electrocardiographic examinations

Electrocardiographic recordings were performed using a 3-channel ECG (Edan Instruments, Inc., VE-300, China) at paper speed of 25 mm/s and calibration of 10 mm/mV. Thermosensitive ECG paper with a total width of 50 cm and a recording width of 80 mm was used. One small square on the horizontal and vertical axis was equal to 0.04 s and 0.1 mV, respectively. The recordings were obtained for all the standard bipolar limb leads (leads I, II, and III) and unipolar augmented limb leads (leads aVR, aVL, and aVF). During recording, all dogs were restrained manually in the right lateral recumbency without any tranquilizer or anesthetic drug. Lead II was considered as the typical wave to analyze the depolarization vector. Amplitude and duration of P waves, QRS complexes, and T waves were measured together with PR and QT interval. HR was evaluated using R-R interval. The triangulation method was used to calculate the QRS complex of leads I and III to obtain MEA [20].

### Statistical analysis

Generalized linear models were used for analysis. The effects of age and body weight were adjusted for covariate variable, and grouping of animals was based on the effects of sex and breed (equation 1). p<0.05 was considered as statistically significant. SAS software university edition (SAS, Inc., Cary, NC, USA) was used for data analysis.


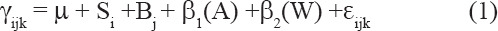


Where:

γ_ijk_ is observation of parameters, µ is the overall mean, S_i_ is the effect of sex, B_j_ is the effect of breed, β_1_(A) is the covariate of age, β_2_(W) is the covariate of body weight, and ε_ijk_ is the residual error.

## Results

**Figure-1:**
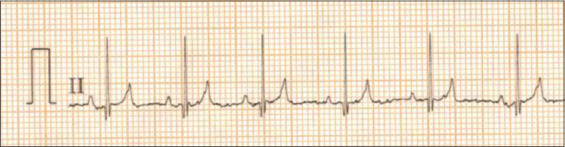
Normal electrocardiograph (Lead-II, 25 mm/s and 10 mm/mV) of Labrador dog.

**Figure-2:**
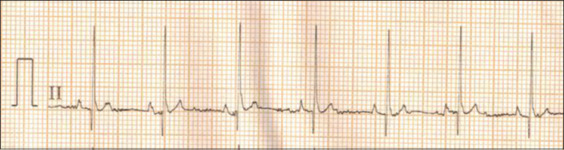
Normal electrocardiograph (Lead-II, 25 mm/s and 10 mm/mV) of German Shepherd dog.

**Figure-3:**
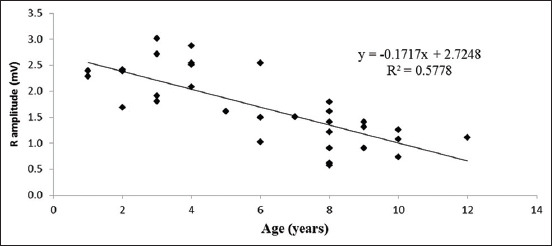
Scatter plot of the correlation between R amplitude and age showing a significant decrease in R amplitude with increase of age in dogs.

ECG parameters were expressed as least square mean ± standard error. ECG recordings at lead II in Labrador Retrievers and German Shepherds are presented in Figures-1 and 2, respectively. In this study, ECG parameters were not significantly associated with breed and sex (Tables-1 and 2). However, QRS duration changed depending on the breed (p<0.1). Labrador Retrievers (0.04±0.005 s) had lower QRS duration than German Shepherds (0.05±0.005 s) (Table-1). PR interval was influenced by sex (p<0.1), with females (0.13±0.005 s) having higher PR interval than males (0.11±0.008 s) (Table-2). ECG parameters were not significantly associated with age and body weight of the dogs, except that R wave amplitude had a significantly decreased correlation with the increase of age (p<0.05) (Figure-3).

**Table-1:** The effect of breed on electrocardiographic parameters of dogs.

ECG parameters	Breed		p-value

	Labrador Retriever	German Shepherd	
HR (beats/min)	92.25±4.651	97.23±4.895	0.501
P wave amplitude (mV)	0.21±0.023	0.19±0.025	0.648
P wave duration (s)	0.04±0.003	0.05±0.004	0.262
PR interval (s)	0.12±0.006	0.12±0.006	0.544
R amplitude (mV)	1.78±0.142	1.64±0.149	0.547
QRS duration (s)	0.04±0.005	0.05±0.005	0.058
QT interval (s)	0.21±0.007	0.19±0.007	0.162
T wave amplitude (mV)	0.38±0.064	0.23±0.067	0.149
T wave duration (s)	0.067±0.0068	0.067±0.0071	0.970
MEA (degrees)	78.30±4.320	86.61±4.200	0.213

ECG=Electrocardiograph, HR=Heart rate, MEA=Mean electrical axis

**Table-2:** The effect of sex on electrocardiographic parameters of Labrador Retriever and German Shepherd dogs.

ECG parameters	Sex		p-value

	Female	Male	
HR (beats/min)	98.17±3.735	91.32±5.895	0.382
P wave amplitude (mV)	0.20±0.019	0.20±0.031	0.921
P wave duration (s)	0.05±0.003	0.05±0.004	0.728
PR interval (s)	0.13±0.005	0.11±0.008	0.059
R amplitude (mV)	1.66±0.114	1.76±0.180	0.659
QRS duration (s)	0.05±0.004	0.04±0.006	0.531
QT interval (s)	0.21±0.006	0.19±0.009	0.213
T wave amplitude (mV)	0.31±0.051	0.30±0.081	0.907
T wave duration (s)	0.07±0.005	0.07±0.009	0.809
MEA (degrees)	80.44±3.460	84.46±5.110	0.565

ECG=Electrocardiograph, HR=Heart rate, MEA=Mean electrical axis

## Discussion

ECG parameters for different breeds of trained dogs such as Labrador Retriever, German Shepherd, and Golden Retriever have been reported [16]. However, the correlations of ECG values with sex, age, and body weight were not clearly understood. Thus, this study was established to examine them in detail. Reference values of ECG parameters for Labrador Retrievers and German Shepherds in this study were found to be in agreement with earlier reports [16,18,21].

In this study, HR ranged from 60 to 130 beats/min, similar to a previous report [22]. There were no significant differences in HR between Labrador Retrievers and German Shepherds, as reported earlier [16]. P wave is the first deflection on ECG, corresponding to atrial depolarization, and spreads from sinoatrial to AV node. In this study, we found that positive P wave is the deflection in leads I, II, III, and aVF and negative P waves are the deflection in leads aVR and aVL. No significant difference was found in P wave amplitude between Labrador Retrievers and German Shepherds, but earlier reports revealed that it was slightly higher in Labrador Retrievers compared with German Shepherds [16,21]. Higher P wave amplitude in Labrador Retrievers may be due to stress during ECG recording [17]. There was no significant effect of sex on P wave amplitude, similar to earlier reports [21]. Sex influenced PR interval (the time from the start of atrial depolarization wave traveling through the AV node to the bundle of His) (p<0.1). The variation in PR interval may be due to sinus arrhythmia [22].

In this study, the Q wave of the QRS complex, which is initially the first part of the ventricles to depolarize through the ventricular septum, was negative deflection in leads I, II, III, aVL, and aVF, whereas positive deflection in aVR. This is contradictory to Mukherjee *et al*. [16], who found that aVR and aVL were positive deflection and Gugjoo *et al*. [21] who found an absence of Q wave in aVR and aVL. However, Avizeh *et al*. [17] reported the presence of Q wave in almost all leads.

R wave reflects the depolarization of the main mass of the ventricles and produces a large deflection due to a large mass of ventricular muscle [20]. This study found that R wave amplitude was not significantly different between the two breed groups (Labrador Retriever = 1.78±0.142 mV and German Shepherd = 1.64±0.149 mV). This finding was not supported by Mukherjee *et al*. [16] but was in accordance with Gugjoo *et al*. [21] and Spasojevic Kosic *et al*. [18]. Variation in R wave amplitude may be due to different breed-dependent sizes of dogs [1]. In this study, the effect of age leads to altered R amplitude; a significant decrease in R wave amplitude increases by age, according to Spasojevic Kosic *et al*. [18]. Increase in age may result in the disturbance of impulse conduction and changes in ventricular depolarization in dogs.

In our study, QRS duration (the period of ventricular depolarization) was not significantly different between Labrador Retrievers and German Shepherds, similar to earlier reports [16]. Breed influencing QRS duration (p<0.1) may be due to a varying mass of cardiac muscle in the ventricular chambers in different breeds. However, values of QRS duration of both dog breeds were within the reference range. QT interval represents the entire period of ventricular depolarization and repolarization, which was found to be in accordance with earlier reports [16,18,21].

T wave amplitude and duration are the repolarization of the ventricular myocardium and were found to be within the normal range in our study, as reported by earlier studies [16,18,21]. We also found that T wave can be positive or negative deflection in lead II of ECG, which has been found in normal dogs [23].

MEA represents the total sum of electrical vector directions produced by the action potentials of individual ventricular myocardial cells. In this study, there was no significant difference in MEA with breed or sex, similar to the previous reports [21] but contrary to Mukherjee *et al*. [16], who reported that there was a significant variation in MEA found between Labrador Retrievers and German Shepherds. In our investigation, the values of MEA were between +60° and +120°. This variation of MEA might due to varying thoracic shapes in different breeds [24].

## Conclusion

This study established that different ECG parameters were within the normal range in both Labrador Retrievers and German Shepherds. There was a significant difference in R wave amplitude on the effect of age. However, no significant differences were found in ECG parameters on the effects of breed, sex, or body weight.

## Authors’ Contributions

WK designed the study and analyzed the data. ST, SY, SI, and WL recorded and analyzed the data. WL coordinated the study, wrote, and revised the manuscript. All authors read and approved the final manuscript.

## References

[1] Tilley L.P, Cann C.C (1992). Essentials of canine and feline electrocardiography. Interpretation and Treatment.

[2] Daniel R.C, Moodie E.W (1979). Relationship between plasma calcium and QT interval of electrocardiogram in dairy cows. J. Dairy Sci.

[3] Harvey A.M, Faena M, Darke P.G, Ferasin L (2005). Effect of body position on feline electrocardiographic recordings. J. Vet. Intern. Med.

[4] Pradhan R.R, Mahapatra A.P.K, Mohapatra S, Jyotiranjan T, Kundu A.K (2017). Electrocardiographic reference values and configuration of electrocardiogram waves recorded in black Bengal goats of different age groups. Vet. World.

[5] Skarda R.T, Muir W.W, Milne D.W, Gabel A.A (1976). Effects of training on resting and postexercise ECG in standardbred horses, using a standardized exercise test. Am. J. Vet. Res.

[6] Winter R.L, Bates R.M (2018). Retrospective evaluation of notched QRS complexes in dogs:85 cases. J. Vet. Cardiol.

[7] Noszczyk-Nowak A, Michalek M, Kaluza E, Cepiel A, Paslawska U (2017). Prevalence of arrhythmias in dogs examined between 2008 and 2014. J. Vet. Res.

[8] Jung S.W, Sun W, Griffiths L.G, Kittleson M.D (2016). Atrial fibrillation as a prognostic indicator in medium to large-sized dogs with myxomatous mitral valvular degeneration and congestive heart failure. J. Vet. Intern. Med.

[9] Wright K.N, Atkins C.E, Kanter R (1996). Supraventricular tachycardia in four young dogs. J. Am. Vet. Med. Assoc.

[10] Lee P.M, Brown R.H.T (2019). Establishing 24-hour holter reference intervals for clinically healthy puppies. Res. Vet. Sci.

[11] Moise N.S, Gilmour R.F, Riccio M.L, Flahive W.F (1997). Diagnosis of inherited ventricular tachycardia in German shepherd dogs. J. Am. Vet. Med. Assoc.

[12] Dae M.W, Lee R.J, Ursell P.C, Chin M.C, Stillson C.A, Moise N.S (1997). Heterogeneous sympathetic innervation in German shepherd dogs with inherited ventricular arrhythmia and sudden cardiac death. Circulation.

[13] Kornreich B.G, Moise N.S (1997). Right atrioventricular valve malformation in dogs and cats:An electrocardiographic survey with emphasis on splintered QRS complexes. J. Vet. Intern. Med.

[14] Kovacevic A, Sastravaha A (2007). Clinically silent atrial dissociation in a dog. J. Vet. Cardiol.

[15] Westling J, Westling W, Pyle R.L (2008). Epidemiology of atrial fibrillation in the dogs. Intern. J. Appl. Res. Vet. Med.

[16] Mukherjee J, Das P.K, Ghosh P.R, Banerjee D, Sharma T, Basak D, Sanyal S (2015). Electrocardiogram pattern of some exotic breeds of trained dogs:A variation study. Vet. World.

[17] Avizeh R, Papahn A.A, Ranjbar R, Rasekh A.R, Molaee R (2010). Electrocardiographic changes in the littermate mongrel dogs from birth to six months of life. Iran. J. Vet. Res.

[18] Kosic L.S, Trailovic D.R, Krstic N (2017). Age-dependent electrocardiographic and echocardiographic changes in German shepherd dogs. Iran. J. Vet. Res.

[19] Aleixo A.S.C, Alfonso A, Oba E, de Souza F.F, Cruz R.K.S, Fillippi M.G, Chiacchio S.B, Tsunemi M, Lourenco M.L.G (2017). Scaling relationships among heart rate, electrocardiography parameters, and body weight. Top. Companion Anim. Med.

[20] Martin M (2007). Small Animal ECGs:An Introductory Guide.

[21] Gugjoo M.B, Hoque M, Saxena A.C, Zama M.M (2014). Reference values of six-limb-lead electrocardiogram in conscious Labrador retriever dogs. Pak. J. Biol. Sci.

[22] Hanton G, Rabemampianina Y (2006). The electrocardiogram of the beagle dog:Reference values and effect of sex, genetic strain, body position and heart rate. Lab. Anim.

[23] Willis R (2018). Guide to Canine and Feline Electro-cardiography.

[24] Carnabuci C, Tognetti R, Vezzosi T, Marchesotti F, Patata V, Domenech O (2019). Left shift of the ventricular mean electrical axis in healthy Doberman Pinschers. J. Vet. Med. Sci.

